# Oral Cancer Incidence Among Adult Males With Current or Former Use of Cigarettes or Smokeless Tobacco: Population-Based Study

**DOI:** 10.2196/51936

**Published:** 2024-11-06

**Authors:** Brendan Noggle, Hui Cheng, Mohamadi Sarkar

**Affiliations:** 1 Altria Client Services LLC, 601 E Jackson St, Richmond, VA, 23219, United States, 1 (804) 484-8222

**Keywords:** tobacco harm reduction, oral cancer, smokeless tobacco, smoking, cancer epidemiology, cancer registry, population-based study, oral cancer incidence, cancer cases

## Abstract

**Background:**

Tobacco use has been identified as a risk factor for oral cancer worldwide. However, relative oral cancer incidence among adults who smoke cigarettes, use smokeless tobacco products (ST), have transitioned from cigarettes to ST, quit cigarettes and/or ST (“quitters”), or never used tobacco has not been well studied.

**Objective:**

We aim to present population-based oral cancer incidence rates for adults who smoke cigarettes, use ST, are former smokers who now use ST, or quit.

**Methods:**

We estimated cross-sectional incidence rates and incidence rate ratios (IRRs) using data from statewide cancer registries (Colorado, Florida, North Carolina, and Texas) and population counts derived from national surveys using combined data from 2014‐2017. A random-effect meta-analysis approach was used to summarize estimates among these groups, based on multiple imputation-based IRR estimates by state and age group while considering potential heterogeneity.

**Results:**

A total of 19,536 oral cancer cases were identified among adult males 35 years and older in the study geographies and period. The oral cancer incidence rate among adults who smoke was significantly higher than the ST group (2.6 times higher, 95% CI 2.0‐3.3, *P*<.001), 3.6 (95% CI 3.2‐4.1, *P*<.001) times higher than the never users, and 2.4 (95% CI 1.8‐3.1, *P*<.001) times higher compared to former smokers who now use ST. The IRR among the ST group relative to never users was 1.4 (95% CI 1.1‐1.9, *P=*.02). The IRR between former smokers who now use ST and those who quit was 1.4 (95% CI 1.0‐2.1, *P=*.08).

**Conclusions:**

Findings from this population-based study with a large number of oral cancer cases support significantly high oral cancer incidence among adults who smoke and a lower risk of oral cancer incidence among never users, quitters, users of ST, and former smokers who now use ST compared to cigarettes. Future studies with detailed control of tobacco history and other relevant confounders are needed to confirm these findings.

## Introduction

Oral cancer is one of the most common cancers worldwide, particularly among males [1]. Oral cancer comprises almost 3% of new cancers in the United States, with incidence rates rising in the last decade [2]. The global incidence of cancers of the lip and oral cavity is estimated at 4.1 cases per 100,000 people, however, there is wide variation across the globe [1]. In some Asian-Pacific countries, the incidence of oral cancer ranks among the 3 top cancers [3]. Oral cancer or mouth cancer includes cancers of the lip, oral cavity, and the pharynx (hence sometimes referred to as oropharyngeal cancer) [4]. Oral cancers most commonly develop on the tongue and other parts of the mouth [5]. Oral cancer is more common in men and in older people and varies strongly by socioeconomic condition. Tobacco, alcohol, and areca nut (betel quid) use are among the leading causes of oral cancer [6]. In North America and Europe, human papillomavirus (HPV) infections are responsible for a growing percentage of oral cancers [7,8].

The published evidence regarding the association of oral cancer with tobacco use has primarily focused on combustible cigarettes and smokeless tobacco (ST) products. Previous studies have shown a consistent association between cigarette use and elevated risks of oral cancer [9-12]. For example, a meta-analysis by Gandini et al found substantially elevated risk of oral cavity cancer (relative risk 3.43, 95% CI 2.37‐4.94, based on 14 studies) and pharyngeal cancer (relative risk 6.76, 95% CI 2.86‐15.98, based on 7 studies) among people who currently smoke cigarettes. With a focus on mortality from oral cancer, Rostron [13] reported 10.89 times higher risk of oral cancer mortality risk among males and 5.08 times higher mortality risk among females who smoke cigarettes compared to those who never smoke cigarettes. Similarly, Coleman identified a 4.84 times higher risk of oral and oropharyngeal cancer mortality among current smokers after adjusting for air pollution and other covariates [14]. Additionally, Fisher [15] reported a 6.33 (95% CI 1.46‐27.38) times higher oral cancer mortality risk among people who currently smoke cigarettes (and have never used ST) compared to those who never used any tobacco products.

ST, a Group 1 carcinogen as classified by the International Agency for Research on Cancer [16], has also been associated with oral cancer, with notable regional differences. ST products vary widely in type and composition around the world, and there are marked regional differences in patterns of consumption. These differences may explain the substantial differences between the health risks of different ST products and their associated disease burden across different countries and regions [17-21]. Elevated health risks have been observed in the South Asian and eastern Mediterranean regions [20,22,23], where many common ST products (eg, gutkha, zarda, paan, khaini) contain high levels of carcinogens, notably tobacco-specific nitrosamines and heavy metals, compared to products commonly used in Sweden [24] and the United States [25-27]. For example, the total tobacco-specific nitrosamine levels are 5- to 25-fold higher in Southeast Asian zarda (mean 0.051 mg/g) [25] than in the ST products used in Sweden (mean 0.002 mg/g) and the United States (mean 0.01 mg/g) [28]. Recently, 2 meta-analyses concluded that there is a positive association between ST use and oral cancer worldwide; however, associations varied by geographic region such that studies in North America showed no associations with oral cancer [20,29]. Conversely, 3 US-focused meta-analyses identified a 1.39- [30], 1.65- [31], and 2.6-fold [32] increased oral cancer risk among US ST users compared to nonusers. Of note, these 3 meta-analyses included studies published over a wide time frame, and 2 noted a pattern of decreasing risk estimates over time [30,31]. Past commentary has identified varied definitions of oral cancer type, inconsistent control for smoking, and time frame of studies as contributing reasons for inconsistent results in commonly cited meta-analyses [33]. Importantly, there is sparse data comparing oral cancer incidence among individuals who have stopped smoking and use ST products relative to continued smokers or those who have quit both cigarettes and ST products. Further, many studies among ST users are hampered by small oral cancer case sizes [15,34-37].

The data for this study were collected by population-based cancer registries and provide detailed information on tobacco use and cancer incidence. All US states and many substate jurisdictions actively collect information on tumors that occur within the surveillance area with the goal of providing accurate and timely information on cancer incidence, treatment, and survivorship [38]. Information on cancer cases and treatment collected within hospitals and other medical facilities is consolidated by a state or local cancer registrar, then it is standardized and made available for study [39]. In 2011, some states began collecting enhanced information on tobacco use risk factors including past and current cigarette, ST, and other tobacco use [40]. These large population-based cancer registries allow the combination of oral cancer cases with a valid population base to estimate oral cancer incidence rates among various adult male tobacco use groups. This study is the first to leverage state-based cancer registries to estimate and compare the incidence of oral cancer among adult males who smoke cigarettes, use ST products, are former smokers who now use ST, quit cigarettes and/or ST (“quitters”), and are never users of tobacco products in select US states.

## Methods

### Data Source and Study Population

We used data from state cancer registries, which provided coverage of all cancer cases in the entire state, to identify oral cancer cases. We used data from the Tobacco Use Supplement to the Current Population Survey (TUS-CPS) to estimate the number of individuals in each state based on their tobacco use status. Incidence rates of oral cancer among tobacco use groups were calculated by dividing the number of oral cancer cases (from the state registries) by the population estimates (from TUS-CPS); see the Data Analysis section for additional details.

Cancer registry data were combined from the Colorado (CO), Florida (FL), North Carolina (NC), and Texas (TX) state cancer registries from the years 2014 through 2017. Although cancer registries provide robust data on cancers diagnosed in their jurisdiction, they often lack complete and accurate collection of data on cigarette and ST use. The registries selected for this study are different from other registries because during this time period, they gathered enhanced tobacco use information in addition to the regularly collected cancer incidence and demographic information. This enhanced tobacco use information includes current, never, or former use of cigarettes and/or ST, and some other tobacco use behaviors. Tobacco use risk behavior data was relatively more complete during the study period (>60% of records with tobacco data) than in prior years.

The population denominator in this study is from the available July 2014, January 2015, May 2015, and July 2018 administrations of the TUS-CPS, a nationally representative survey sponsored by the National Cancer Institute as a part of the US Census Bureau’s Current Population Survey. We combined 4 years of case data and 4 years of population data to construct a reasonably accurate incidence rate using population data from the year closest to case data years. TUS-CPS data were weighted for selection probabilities and nonresponse; poststratification factors were applied to balance the sample against the population estimates for each state. Population size of tobacco use/nonuse groups were generated using the weighted counts. Behavioral Risk Factor Surveillance System (BRFSS) estimates were used to replace any zero denominator in the rare event that there was no individual in a state-specific tobacco use group in the TUS-CPS data.

In this study, we focused on the US male population ≥35 years of age because of the limited number of oral cancer cases among individuals younger than 35 years and limited numbers of female ST users, which precluded estimates with reasonable precision when stratified by state and age. Moreover, because >90% of ST users in the United States were males [41], we consider our results generalizable to the majority of the US population of ST users.

### Oral Cancer Definition

We included the following invasive malignant oral tumors as oral cancers based on the International Classification of Diseases for Oncology, Third Edition: lip (codes C000-C009), tongue (C019-C029), salivary gland (C079-C089), floor of mouth (C040-C049), gum and other mouth (C030-C039, C050-C059, C060-C069), nasopharynx (C110-C119), tonsil (C090-C099), oropharynx (C100-C109), hypopharynx (C129, C130-C139), and other oral cavity and pharynx (C140, C142, C148). We excluded lymphoma and hematopoietic histology (9050‐9055, 9140, 9590‐9992) to meet the current Surveillance, Epidemiology, and End Results Program and World Health Organization definition.

### Tobacco Use Status

The state cancer registry data contained variables coding never, current, and former cigarette smoking and ST use (including moist loose or pouched snuff, chewing tobacco, snus, dry snuff) status based on self-reported information when included in the medical records relevant to the cancer diagnosis. In TUS-CPS, information on tobacco use was collected via survey questions about cigarette smoking and ST use (including moist snuff, dip, spit, chew tobacco, or snus). The population data were coded into the same “never,” “current,” and “former” categories as case data. We defined never smokers as individuals who have never smoked at least 100 cigarettes in their lifetime and never ST users as individuals who have never used ST. We defined current users as ever users who responded “everyday” or “some days” when asked whether they smoked or used ST now. Actual survey questions were utilized from the TUS-CPS Questionnaires as described on the website [42].

Using the “never,” “current,” and “former” categories, we combined the cancer cases and the population denominator into the following tobacco use groups: the never cigarette never ST group (Never Cig/Never ST) included individuals who were never users of cigarettes and never users of ST; the cigarette smoking group (Current Cig/Never ST) included individuals who were current users of cigarettes but never users of ST; the ST group (Never Cig/Current ST) included individuals who currently used ST but never cigarettes; the dual user group (Current Cig/Current ST-Dual) included individuals who currently used cigarettes and ST; the former smokers who now use ST group (Former Cig/Current ST) included those who were former smokers (last used cigarettes over 12 months ago) and currently used ST; and the former smoker former ST group (Former Cig/Former ST), also referred to as “quitters,” irrespective of other tobacco use. In this study, we considered the former smokers who now use ST group as individuals who smoked in the past, stopped smoking, and now currently use ST, although the temporal nature of the tobacco use transition was not precisely reported. Other tobacco states and possible transitions were not included in this analysis.

### Data Analysis

Oral cancer incidence rates were calculated by dividing the number of oral cancer cases from the state cancer registry by weighted population counts estimated from TUS-CPS for each tobacco user group. Incidence rate ratios (IRRs) were calculated using Poisson regression for each age group (35‐44, 45‐54, 55‐64, 65‐74, and ≥75 years) and each state of residence at diagnosis. A random-effect meta-analysis approach was used to summarize state- and age-specific estimates while taking into account potential heterogeneity [43].

Missing data on cigarette and ST use in state cancer registries ranged from 21% in CO to 37% in TX. In order to account for missing values in tobacco use variables that would lead to unnaturally low rates, we assumed the rate of cancer incidence by state and age group was the same among the records with and without tobacco data. Using this assumption, we conservatively weighted the number of oral cancer cases among records with tobacco use data at a proportion equal to the amount of missing values in the tobacco use variable by state and age to allow for incidence rate comparisons between tobacco use groups.

Once rates were constructed, we took 2 approaches during data analysis and present results from each. First, as described above, we inflated the number of oral cancer cases at a proportion equal to the amount of missing values in the tobacco use variable by state and age to allow for incidence rate comparisons between tobacco use groups. This provided incidence rates for each tobacco use group adjusted for state and age. Second, we used a multiple imputation approach [44]. Multiple imputation was conducted to understand the potential impact of missing data on estimates. Variables used in multiple imputation for all states include cancer site, year of diagnosis, age at diagnosis, sex, and race/ethnicity. Other states shared additional information that could be used during multiple imputation. For example, the states of TX and CO included the degree of malignancy and spread in the body, which were also included in the multiple imputation. Additionally, TX provided county of residence, poverty level, and cancer grade. A total of 10 imputations were generated (seed number=212,215). Augmented regression was used to address any perfect prediction by adding a few observations with small weights to the data during estimation to avoid perfect prediction. Analysis was conducted using SAS 9.4 (SAS Institute) and Stata 16 (StataCorp LLC).

### Ethical Considerations

This study was conducted in accordance with the Declaration of Helsinki and its amendments, and the data analysis protocol was approved by Advarra, an independent Institutional Review Board (Pro00042038). The written informed consent of the participants was waived by Advarra. Permission to use the data was obtained from individual state cancer registries and is governed by data use agreements. Privacy and confidentiality protections are in place and study data did not include personal identifiers.

## Results

### Sample Description

A total of 36,270 oral cancer cases among adults 35 years and older were included in this study, with 73.5% (n=26,666) of cases among males. Of these cases in the registries, almost three-quarters of cases, 73.3%, had tobacco use data, to yield a final sample of 19,536 oral cancer cases among males 35 years and older. The distribution among the groups identified were as follows: Never Cig/Never ST=32.4% (n=6325); Current Cig/Never ST=24.4% (n=4770); Never Cig/Current ST=1% (n=190); Current Cig/Current ST-Dual=0.8% (n=153); Former Cig/Current ST=0.7% (n=136); Former Cig/Former ST=2.3% (n=443). Other tobacco use combinations make up the remaining 38.5% (n=7519), with 96% (n=7218) of that remainder being former smokers who have not used ST. Case distribution by state, age, ethnicity, race, and tobacco group are included in Table 1. The cancer registries are located in geographically diverse areas of the United States, with FL and TX contributing the most cases to this analysis. The percentages of total cases were highest in the 55‐64 years age group. Case ethnicity and race varied across states at a level that generally reflected local population demographics. For example, TX and FL had a higher proportion reporting Hispanic or Latino origin than other study states and NC had a proportionally higher Black or African American population. Most cases were White, non-Hispanic.

**Table 1.** Demographics and oral cancer case description among males aged ≥35 years, 2014‐2017. Study case data are from respective state cancer registries and are abstracted from the patient medical record. “Never” tobacco use refers to evidence of never use of cigarettes and/or ST, “current” use refers to evidence of use at time of diagnosis, and “former” use refers to evidence of use in the past but nonuse at time of diagnosis. “Other” tobacco users are excluded from further analysis and tabulations.

Characteristic		Colorado, n (%)	Florida, n (%)	North Carolina, n (%)	Texas, n (%)	Overall, n (%)
**Cases**						
	Oral cancers	1897 (100)	11,525 (100)	4241 (100)	9003 (100)	26,666 (100)
	Oral cancers with tobacco data	1504 (79.3)	8779 (76.2)	3542 (83.5)	5711 (63.4)	19,536 (73.3)
**Age group (years)**						
	35‐44	71 (4.7)	275 (3.1)	148 (4.2)	269 (4.7)	763 (3.9)
	45‐54	258 (17.2)	1446 (16.5)	688 (19.4)	1053 (18.4)	3445 (17.6)
	55‐64	528 (35.1)	3000 (34.2)	1257 (35.5)	1952 (34.2)	6737 (34.5)
	65‐74	421 (28)	2548 (29)	952 (26.9)	1611 (28.2)	5532 (28.3)
	≥75	226 (15)	1510 (17.2)	497 (14)	826 (14.5)	3059 (15.7)
**Ethnicity and race**						
	Hispanic	107 (7.1)	999 (11.4)	69 (2)	713 (12.5)	1888 (9.7)
	White non-Hispanic	1316 (87.5)	7001 (79.8)	2899 (81.9)	4430 (77.6)	15,646 (80.1)
	Black non-Hispanic	47 (3.1)	586 (6.7)	493 (13.9)	382 (6.7)	1508 (7.7)
	Other/unknown	34 (2.3)	193 (2.2)	81 (2.3)	186 (3.3)	494 (2.5)
**Tobacco group^a^**						
	Never Cig/Never ST	503 (33.4)	2641 (30.1)	865 (24.4)	2316 (40.6)	6325 (32.4)
	Current Cig/Never ST	315 (20.9)	2167 (24.7)	1024 (28.9)	1264 (22.1)	4770 (24.4)
	Never Cig/Current ST	24 (1.6)	36 (0.4)	45 (1.3)	85 (1.5)	190 (1)
	Current Cig/Current ST-Dual	14 (0.9)	50 (0.6)	41 (1.2)	48 (0.8)	153 (0.8)
	Former Cig/Current ST	14 (0.9)	24 (0.3)	48 (1.4)	50 (0.9)	136 (0.7)
	Former Cig/Former ST	34 (2.3)	178 (2)	129 (3.6)	102 (1.8)	443 (2.3)
	Other	600 (39.9)	3683 (42)	1390 (39.2)	1846 (32.3)	7519 (38.5)

a Cig: cigarette; ST: smokeless tobacco. Group descriptions are as follows: Never Cig/Never ST were never users of cigarettes and never users of ST; Current Cig/Never ST were current users of cigarettes but never users of ST; Never Cig/Current ST currently used ST but never cigarettes; Current Cig/Current ST-Dual were current users of cigarettes and ST; Former Cig/Current ST were former smokers (last used cigarettes over 12 months ago) and currently used ST; Former Cig/Former ST stopped using both cigarettes and ST.

The population base was calculated for each state, age group, and tobacco user group combination using TUS-CPS. Seven of these estimates could not be calculated due to a lack of tobacco user respondents and they were replaced with BRFSS estimates, including 5 from the relatively smaller groups of Current Cig/Current ST-Dual users (2 in CO, 2 in FL, 1 in TX), 1 from Former Cig/Current ST users (CO), and 1 from Never Cig/Current ST users (CO).

### Incidence Rates

Oral cancer incidence rates (Table 2) among males ≥35 years old were highest among the current smoking groups and lowest among current nonsmoking groups. The overall incidence rate in the Never Cig/Never ST group was 22.1 per 100,000 (95% CI 21.5‐22.6). The overall incidence rate in the Never Cig/Current ST group (20.6 per 100,000, 95% CI 18.3‐23.3) was not significantly different from never users (*P*=.29) and was significantly lower than the Current Cig/Never ST group (74.0 per 100,000, 95% CI 71.9‐76.2; *P*<.001) and Current Cig/Current ST-Dual group (40.6 per 100,000, 95% CI 35.4‐46.6; *P*<.001). The overall incidence rates among Former Cig/Current ST and Former Cig/Former ST (quitters) were not significantly different between the 2 groups, at 18.8 per 100,000 (95% CI 16.3‐21.8) and 18.0 per 100,000 (95% CI 16.6‐19.6; *P*=.60), respectively. Incidence rates were generally consistent between states, with limited differences being observed within the subgroups with smaller sample sizes (ie, Former Cig/Former ST, Former Cig/Current ST, Current Cig/Current ST-Dual).

**Table 2.** Oral cancer incidence rates per 100,000 among males aged ≥35 years by tobacco use status. Rates by state were calculated by Poisson regression. State-specific estimates were adjusted for age group and the overall estimate was adjusted by state and age group.

Group^a^	Colorado, rate (95% CI)	Florida, rate (95% CI)	North Carolina, rate (95% CI)	Texas, rate (95% CI)	Overall, rate (95% CI)
Never Cig/Never ST	22.7 (20.8‐24.7)^c^	23.1 (22.2‐24)^b^^,c^	18.9 (17.7‐20.2)^c^	24.6 (23.7‐25.5)^c^	22.1 (21.5‐22.6)^c^
Current Cig/Never ST	81.6 (73.4‐90.7)^b^^,c^	86.1 (82.6‐89.8)^b^^,c^	83.0 (77.9‐88.5)^b^^,c^	65.2 (62.1‐68.4)^b^^,c^	74.0 (71.9‐76.2)^b^^,c^
Never Cig/Current ST	22.7 (15.8‐32.5)^c^	17.3 (12.9‐23.1)^c^	16.8 (12.9‐22.1)^c^	24.0 (20.2‐28.5)^c^	20.6 (18.3‐23.3)
Current Cig/Current ST-Dual	41.6 (26‐66.6)^b^^,c^	73.3 (57.4‐93.6)^b^^,c^	28.6 (21.6-37.9)^b^	36.7 (29.2‐46.1)^b^^,c^	40.6 (35.4‐46.6)^b^^,c^
Former Cig/Current ST	14.0 (8.8‐22.3)^c^	12.7 (8.9‐18)^c^	23.2 (17.9‐30)	22.0 (17.6‐27.4)^c^	18.8 (16.3‐21.8)
Former Cig/Former ST	7.5 (5.5‐10.1)^b^	29.7 (26.1-33.8)^b^	25.7 (21.9-30.1)^b^	12.6 (10.8‐14.7)^b^	18.0 (16.6‐19.6)

a Cig: cigarette; ST: smokeless tobacco. Group descriptions: Never Cig/Never ST - never used cigarettes or ST; Current Cig/Never ST - current cigarette users, never used ST; Never Cig/Current ST - current ST users, never used cigarettes; Current Cig/Current ST-Dual - current users of both; Former Cig/Current ST - former smokers (before 12 months) and current ST users; Former Cig/Former ST - former users of both

b Significantly different (*P*<.05) rates compared to the Never Cig/Current ST group.

c Significantly different rates (*P*<.05) compared to the Former Cig/Former ST group.

### Incidence Rate Ratios

**Figure 1.**
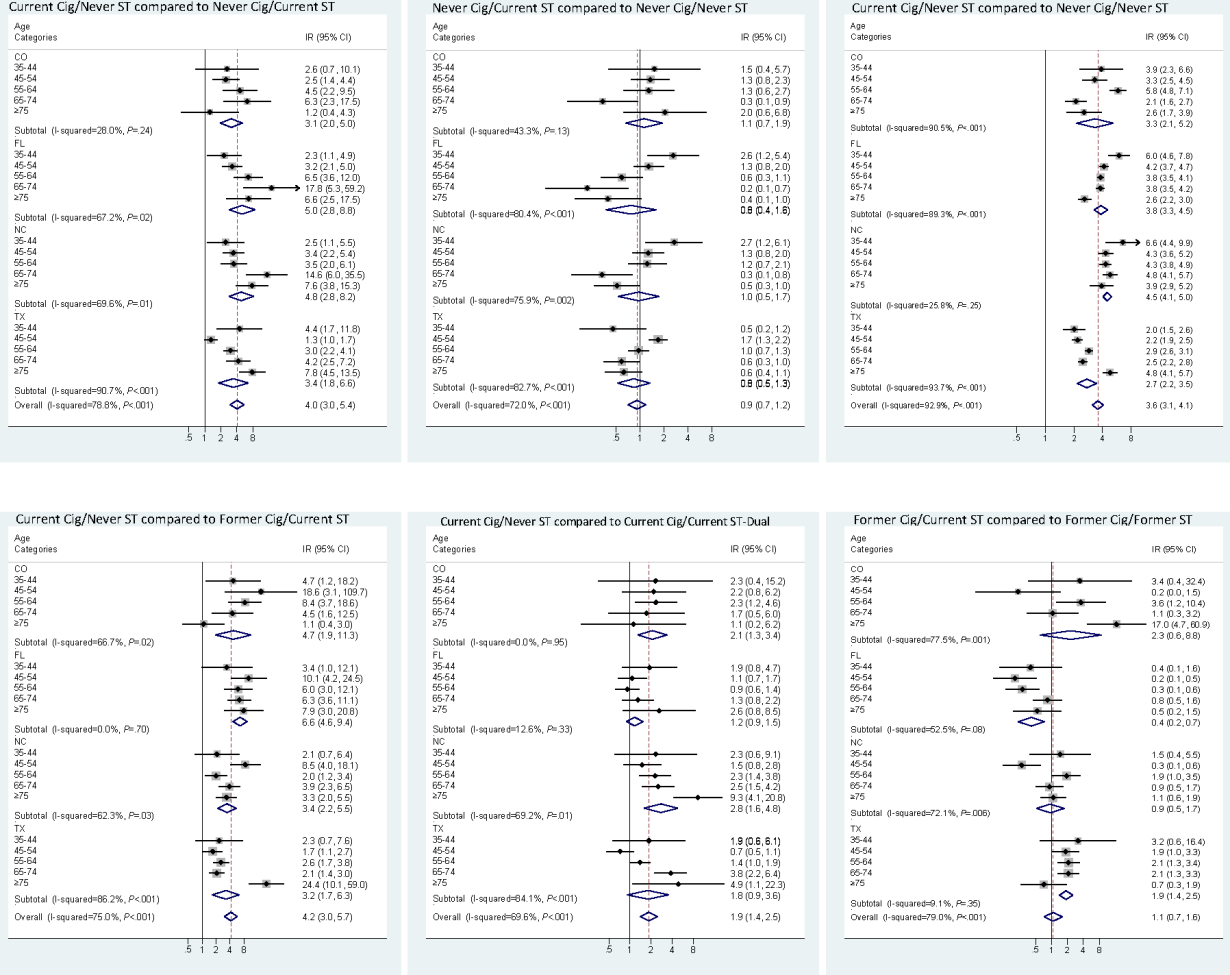
Meta-analysis of incidence rate ratios based on tobacco use status. (A) Current Cig/Never ST group compared to Never Cig/Current ST. (B) Never Cig/Current ST group compared to Never Cig/Never ST. (C) Current Cig/Never ST group compared to Never Cig/Never ST. (D) Current Cig/Never ST group compared to Former Cig/Current ST. (E) Current Cig/Never ST group compared to Current Cig/Current ST-Dual. (F) Former Cig/Current ST compared to Former Cig/Former ST. A value of 1 indicates a null association; a value greater than 1 indicates a positive association; a value less than 1 indicates inverse association, and the horizontal line width reflects the confidence interval. The diamonds indicate the meta-analysis estimate by state and overall and the diamond width represents the confidence interval. The dashed red line indicates the overall estimate for reference. Group descriptions are as follows: Never Cig/Never ST were never users of cigarettes and never users of ST; Current Cig/Never ST were current users of cigarettes but never users of ST; Never Cig/Current ST currently used ST but never cigarettes; Current Cig/Current ST-Dual were current users of cigarettes and ST; Former Cig/Current ST were former smokers (last used cigarettes over 12 months ago) and currently used ST; Former Cig/Former ST stopped using both cigarettes and ST. Cig: cigarette; CO: Colorado; FL: Florida; IR: incidence ratio; NC: North Carolina; ST: smokeless tobacco; TX: Texas.

Using the first approach without multiple imputation (Figure 1), the combined oral cancer incidence rate for the Current Cig/Never ST group was significantly higher, 4.0 (95% CI 3.0-5.4) times, than the Never Cig/Current ST group and 3.6 (95% CI 3.1-4.1) times higher compared to the Never Cig/Never ST group. The incidence rate among the Current Cig/Never ST group was also significantly higher, 4.2 (95% CI 3.0-5.7) times, compared to the Former Cig/Current ST group. The oral cancer incidence rate for the Never Cig/Current ST group was comparable to the Never Cig/Never ST; the IRR estimate was 0.9 (95% CI 0.7-1.2). The estimated rate among the Current Cig/Never ST group was significantly higher, 1.9 (95% CI 1.4-2.5) times, compared to the Current Cig/Current ST-Dual group. Moreover, the comparable rates between the Former Cig/Current ST and Former Cig/Former ST groups yielded an IRR of 1.1 (95% CI 0.7-1.6).

**Figure 2.**
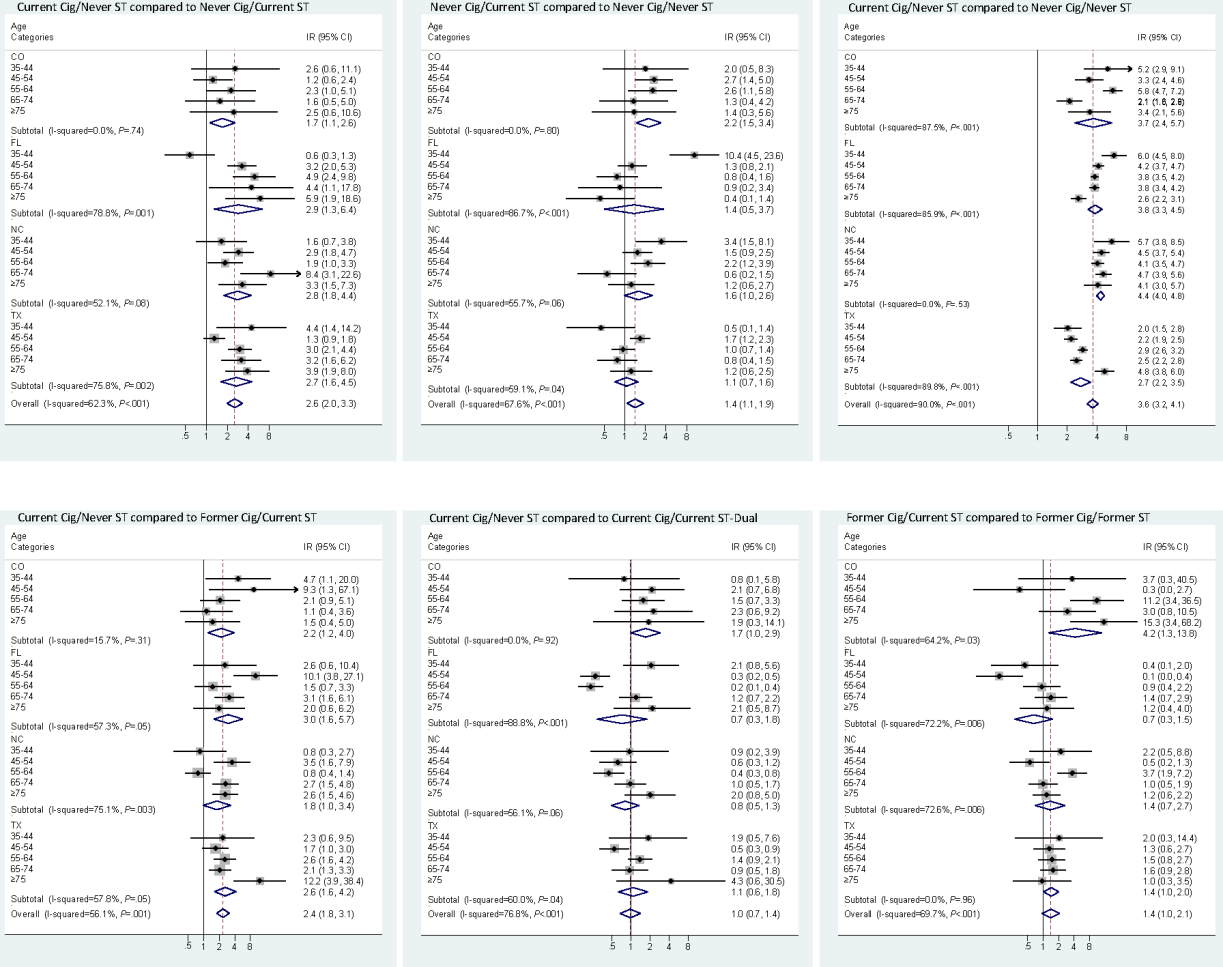
Meta-analysis of incidence rate ratios based on tobacco use status using multiple imputation for missing tobacco use status. (A) Current Cig/Never ST group compared to Never Cig/Current ST. (B) Never Cig/Current ST group compared to Never Cig/Never ST. (C) Current Cig/Never ST group compared to Never Cig/Never ST. (D) Current Cig/Never ST group compared to Former Cig/Current ST. (E) Current Cig/Never ST group compared to Current Cig/Current ST-Dual. (F) Former Cig/Current ST compared to Former Cig/Former ST (ie, quitters). Group descriptions are as follows: Never Cig/Never ST were never users of cigarettes and never users of ST; Current Cig/Never ST were current users of cigarettes but never users of ST; Never Cig/Current ST currently used ST but never cigarettes; Current Cig/Current ST-Dual were current users of cigarettes and ST; Former Cig/Current ST were former smokers (last used cigarettes over 12 months ago) and currently used ST; Former Cig/Former ST stopped using both cigarettes and ST. Cig: cigarette; CO: Colorado; FL: Florida; IR: incidence ratio; NC: North Carolina; ST: smokeless tobacco; TX: Texas.

Results were largely similar when using the multiple imputation approach to address missing tobacco use data (Figure 2). The combined oral cancer incidence rate for the Current Cig/Never ST was significantly higher, 2.5 (95% CI 1.8-3.4) times, compared to Never Cig/Current ST group. Estimates were statistically significant across all states and overall. The combined point estimate for the IRR, when comparing the Never Cig/Current ST group to the Never Cig/Never ST group, was elevated but not statistically significant (combined estimate 1.4, 95% CI 0.97-1.9). The estimate from CO was statistically significant, but it was not for FL, NC, and TX. In contrast to ST, the Current Cig/Never ST group have statistically significant and more elevated risk compared to the Never Cig/Never ST group (combined estimate 3.4, 95% CI 2.9-3.9). Significantly higher oral cancer incidence (combined estimate 2.6, 95% CI 1.9-3.6) was observed for the Current Cig/Never ST group compared to the Former Cig/Current ST group. Oral cancer incidence was comparable between the Current Cig/Never ST and Current Cig/Current ST-Dual groups, with an estimated IRR of 1.0 (95% CI 0.7-1.4). The adjusted IRR for individuals in the Former Cig/Former ST (“quitters”) group relative to the Former Cig/Current ST group was 1.4 (95% CI 0.95-2.1).

## Discussion

### Principal Findings

In this study, we found consistent evidence that individuals who were current users of cigarettes who have never used ST have 2.6 times the oral cancer incidence compared to current users of ST products who have never smoked. In addition, those who used cigarettes in the past and now use ST have lower oral cancer incidence compared to current users of cigarettes. There was a clear oral cancer rate gradient among tobacco use behaviors, where current users of cigarettes have the highest rates, followed by current smokers and ST dual users, with users of ST, never users, and former smoking groups with comparable lower rates. A major strength of the study is the large number of oral cancer cases obtained from state cancer registries, which contained the vast majority, if not all, of oral cancer cases from 4 geographically diverse states across the United States (ie, >19,000 cases in this study), which allowed for robust estimation and enabled specific analysis by tobacco use status (eg, dual users, former smokers who now use ST, and quitters), age group, and state, compared to many previous studies (eg, [35,36,45,46]).

Findings from our analysis on incidence rates are generally consistent with previous reports, including studies from the United States reporting higher relative risks with smoking cigarettes [9,11-14] compared to estimates related to ST use [20,29-32]. For example, US studies consistently show a 3.4- to 10.9-fold elevated oral cancer incidence risk with cigarette smoking relative to never smoking [9,11-14]. In comparison, we note that oral cancer risk estimates for ST product use in the United States have been variable, with some showing nonsignificant associations and others showing an elevated risk; however, they do consistently show mouth cancer risk estimates that are lower than those of cigarette smoking [20,29-32]. Boffetta and colleagues [32] summarized estimates from 9 studies conducted in the United States and found a relative risk of 2.6 (95% CI 1.3-5.2) for oral cancer among ever users of ST compared to nonusers. In another meta-analysis (2009), Lee and Hamling reported a statistically significant risk of oral cancer among people who use ST compared to nonusers after adjusting for smoking (relative risk 1.65, 95% CI 1.22-2.25); however, the differences were not statistically significant when additionally adjusting for alcohol use (relative risk 1.04, 95% CI 0.80-1.35) [31]. Moreover, 2 meta-analyses of more recent epidemiological studies showed no difference in risk among ST product users compared to nonusers [20,29].

This study included recently diagnosed oral cancer cases in order to represent risks associated with more contemporary US tobacco use behaviors and used consistent methodology to construct and compare oral cancer incidence based on a large number of oral cancer cases and population counts from national surveys. These estimates provided a clear and direct comparison of average oral cancer risk between smokers, ST users, former smokers who now use ST, and cigarette and ST quitters. We further demonstrated the consistency of our results by stratifying across age group and state strata. The point estimates indicated a higher incidence of oral cancer in the current cigarette group compared to the ST group across all strata and most estimates were statistically significant at a *P*<.05 level. By using a random-effect meta-analytic approach, we were able to summarize incidence ratio estimates while taking into account any heterogeneity across strata (eg, differences in sample sizes); the meta-analytic incidence ratio estimate was highly robust. Therefore, when considering the overall published literature and our findings, the evidence consistently and clearly indicates that oral cancer risks are substantially higher among adults who smoke cigarettes than adults who use ST products or have quit cigarettes and ST.

This study provides updated population-based estimates of oral cancer risk among ST users based on contemporary ST use behaviors. Although ST products have been used in the US population for almost a century and oral cancer risks have been investigated by others (eg, [31,32,47]), ST products and use patterns have changed over time. For example, studies that included early ST products such as dry snuff use among women, from more than 40 years ago, tended to produce higher relative risk estimates [48] as compared to more recent studies when moist ST was the dominant ST product. Some studies were conducted in specific populations (eg, female Appalachian snuff users [48], agricultural workers [45]), which may not be generalizable to the larger US population; thus, the contemporary analysis presented here adds to the scientific evidence. Moreover, our study presents a unique analysis of more than 19,000 oral cancer cases, which further adds to the body of evidence.

In line with inconsistent evidence on the risk of oral cancer associated with use of ST in the existing literature (eg, [32,36,46]), we found variations in incidence ratio estimates across age groups and states in this study—some estimates were negative, some were positive, and many were null. This finding is not surprising given that the etiology of oral cancer is complex, and some potential confounders were not controlled for due to a lack of such information, including alcohol consumption and HPV infection. Previous studies have found that users of ST were more likely to be heavier alcohol drinkers [49,50]. To further assess potential differences in alcohol drinking, we compared the prevalence of heavy drinking and past 30-day binge drinking using 2018 BRFSS data of the 4 states included in this study and found no statistically significant differences between male smokers and ST users. Furthermore, the existing evidence points to a positive association between tobacco use and HPV [51]. These positive associations between use of ST and potential confounders might have biased the estimates against the null (ie, overestimation).

In this study, we found that males who used cigarettes in the past and now use ST have a substantial reduction in oral cancer risk (>50%) compared to current smokers. A previous study documented that individuals who were former smokers and current snus users tended to be less likely to have oral cancer compared to those who continued to smoke, although the estimate was of borderline statistical significance at the .05 level (odds ratio 0.43, 95% CI 0.18-1.02), possibly due to the moderate sample size of the study (n=139 snuff users) [52,53]. Results from this study extended findings from the previous study to use of ST with greater statistical precision.

Results from this study should be interpreted in the context of the following limitations. First, this study is observational in nature and cannot provide definitive evidence for causal relationships as information about some potential confounders was not available, including details about cigarette consumption, HPV infection, and alcohol consumption. Further, the ecological design of this study precludes individual level inferences. Future studies with detailed control of tobacco history and other relevant confounders, perhaps collected through surveys linked to medical history, could improve the ability to make inferences. Second, oral cancers take years to develop and are impacted by an interplay of various risk factors, which cannot be fully investigated with the cross-sectional approach of this study. The approach presented here may lay the foundation for future studies with the capability of taking a longitudinal approach (eg, retrospective cohort study design) to provide further insights. Third, cancer registry data contains missing tobacco use information and does not precisely characterize types of ST product used, which could lead to tobacco group misclassification. For example, in the United States, ST use includes moist loose or pouched snuff, chewing tobacco, snus, or dry snuff, and this information was not reported in the cancer registry records. However, given that moist loose or pouched snuff (~80% market share) and chewing tobacco (~18% market share) are the most prevalent ST type used in the United States [54,55], we can reasonably assume that these estimates apply to moist ST, the most predominant form of ST use. We applied “never,” “current,” and “former” use categories to all groups, so there is no differential treatment of numerator and denominator. In this study, we used multiple imputation to mitigate the potential impact of missing values and confounders. Inferences were largely consistent between the 2 approaches and showed elevated incidence of oral cancer in the current cigarette group compared to the never user, former smoker who now uses ST, and ST groups. Different statistical inference was drawn with and without using multiple imputation for 2 comparisons (ie, ST use versus never use and smoking versus dual use), which implies nonrandom missing patterns across tobacco use status. Nonetheless, both approaches produced robust estimates, supporting higher oral cancer incidence among current users of cigarettes when compared to users of ST and compared to former smokers who now use ST, respectively. Fourth, cancer registries do not contain information on frequency, intensity, or duration of tobacco use or detailed time since quitting, which precluded a more refined consideration of tobacco use history, particularly transitions from cigarettes to ST products. Nonetheless, despite the lack of this information, we identified higher oral cancer incidence among current users of cigarettes compared to several other tobacco use groups (eg, never smokers, users of ST, former smokers who now use ST) using a similar user definition as other recent studies that revealed comparable differences in risk [15,47]. Improvements to the medical record to include additional types, categories (such as electronic nicotine delivery systems or other novel tobacco products), volume, and duration of tobacco use can enhance future analyses. Fifth, the ST use prevalence is relatively low in the United States (2.3% of adults [56]), which may have contributed to the small sample size within some of the subgroups and imprecise estimates in some age by state strata. We combined multiple years and states to mitigate the impact of small sample size and note that future studies could combine age groups to calculate state-level oral cancer incidence estimates among females.

Despite these limitations, this study did provide comprehensive statewide coverage of cancer cases across 4 large geographically distant states. Here, we highlight the importance of cancer registries as a tool to gain insights into health outcomes related to tobacco use behavior. Our analysis provides evidence regarding the increase in risk of oral cancer among individuals who smoke and supports existing epidemiology demonstrating that these risks are lower among never and former tobacco users, current ST product users, and former smokers who now use ST products.

### Conclusion

Based on our analysis of the data on >19,000 cases in the United States, we present 3 major conclusions. First, smoking cigarettes is linked to oral cancer risk. Second, quitting tobacco or use of ST products is associated with lower risks of oral cancer than cigarette smoking. Third, those who smoked in the past but now use ST products have lower oral cancer risk compared to those who continue to smoke.

These findings have important public health implications. The US Food and Drug Administration and many in the scientific, medical, and public health community [57-60] have concluded that a continuum of risk exists within tobacco products, combustible cigarettes being the highest and noncombustible products like ST products being far lower. Although quitting all tobacco products is the optimum outcome, according to the harm reduction framework [59], smoking-related morbidity and mortality can be reduced by encouraging adult smokers who are unable or unwilling to quit tobacco to switch to less harmful products. However, despite the evidence presented here and supported by other reports [9,11,20,31,32], millions of adults continue to smoke [56]. One of the reasons is that most (~90%) believe that use of ST products is equal to or more harmful than use of cigarettes [61-64]. The misperceptions regarding the risk differential between cigarettes and ST may be dissuading smokers who are unable or unwilling to quit tobacco from switching to lower risk products like ST [65]. Our findings support existing evidence of higher oral cancer risk among individuals who smoke compared to those who quit or used ST products. However, the vast majority of adult smokers are not aware of this evidence. Improved knowledge of the relative risks of ST and cigarettes could allow adult smokers to make informed decisions regarding the benefits of quitting or switching and successfully reduce the harm from smoking-related diseases.

## Abbreviations

BRFSS: Behavioral Risk Factor Surveillance System
CO: Colorado
FL: Florida
HPV: human papillomavirus
IRR: incidence rate ratio
NC: North Carolina
NC: North Carolina
ST: smokeless tobacco
TUS-CPS: Tobacco Use Supplement to the Current Population Survey
TX: Texas
